# A260 PYOGENIC LIVER ABSCESS FORMATION SIX MONTHS AFTER PERFORATED APPENDICITIS IN AN OTHERWISE HEALTHY CHILD

**DOI:** 10.1093/jcag/gwac036.260

**Published:** 2023-03-07

**Authors:** H Sutton, N Carman

**Affiliations:** Gastroenterology Hepatology and Nutrition, The Hospital for Sick Children, Toronto, Canada

## Abstract

**Background:**

Pyogenic liver abscesses (PLA) in children are uncommon in the developed world. When they are diagnosed, it is often in patients with hepatobiliary cancers, biliary diseases and liver transplants. PLA post perforated appendicitis has been reported as an aetiology in non-immunocompromised children. Reported cases of PLA’s developing as a late complication of perforated appendicitis are rare. The longest time from appendicitis to PLA diagnosis previously reported was four months.

**Purpose:**

To report a rare case of liver abscess formation, post ruptured appendicitis, in a previously healthy and review the recent literature relevant to this topic.

**Method:**

Retrospective chart review through electronic patient records was conducted for our reported case. Literature review of the relevant medical literature was performed.

**Result(s):**

Patient is an 11-year-old female with no significant past medical history apart from a perforated appendix requiring laparotomy for washout and 10 days of antibiotics, 6 months prior to presentation. The patient presented to her local emergency room with 6-week history of right flank pain and 10 days of fever. Abdominal ultrasound (US) identified a 3cm x 3cm x 3cm mass in the right lobe of the liver consistent with an abscess which was confirmed with magnetic resonance imaging (MRI). She was transferred to Toronto Hospital for Sick Children for management and investigation. At the Hospital for Sick Children she was started on Intravenous (IV) cefotaxime and metronidazole. After a repeat US on transfer it was determined the liver abscess was not amenable to drainage. Echinococcosis and Entamoeba serology were negative and neutrophil oxidative burst test (NOBT) test was within normal limits. Liver enzymes never exceeded 2x the upper limit of normal and synthetic function remained intact. After 7 days of IV antibiotics the patient’s inflammatory markers had improved and she had remained afebrile, but repeat US and MRI showed little to no change in abscess size. The decision was made for a prolonged course of IV antibiotics. After another two weeks of IV antibiotics her repeat US showed reduction in abscess size by roughly one third, and she was switched to oral antibiotics, after a total of 28 days, for a further four weeks with ongoing improvement.

**Conclusion(s):**

A review of the literature identified one nationwide population-based analysis in the United States reporting 4075 cases of PLAs, as well as three single centre reviews, from Iran, Tailand and the United Kingdom, reporting 18, 15 and 38 cases of liver abscesses, respectively. Common underlying aetiologies included primary immunodeficiency’s, liver transplantation, hepatobiliary cancers and non-PLA intra-abdominal infections. Managements included IV antibiotics plus/minus drainage when amenable. No cases of liver failure or death were reported.

**Please acknowledge all funding agencies by checking the applicable boxes below:**

None

**Disclosure of Interest:**

None Declared

